# Machine learning algorithms: why the cup occasionally appears half-empty

**DOI:** 10.1038/s41430-024-01529-2

**Published:** 2024-10-23

**Authors:** Richard J. Woodman

**Affiliations:** Discipline of Biostatistics, College of Medicine and Public Health, Flinders University, Adelaide, SA, Australia

**Keywords:** Education, Epidemiology

The burgeoning interest in the use of machine learning (ML) algorithms for prediction has led to vigorous debate on whether, despite all the hype, they genuinely outperform more traditional regression based approaches to prediction [1–3]. The paper “Predicting non-responders to lifestyle intervention in prediabetes: a machine learning approach” by Foppiani et al. [4] in this month’s edition of EJCN highlights both the advantages that ML algorithms can offer, and the common trap of judging performance using overall classification accuracy. For this study, the authors goal was to determine whether a ML approach improves the ability to identify individuals with pre-diabetes who did or did not respond to a 12-month lifestyle intervention. Adequate response was considered a normalisation of blood glucose (<100 mg/dL) and the intervention consisted of a hypocaloric omnivorous Mediterranean style diet plus following the WHO physical activity guidelines. Since predicting patients that will respond to treatment is the basis of precision-medicine [5], the study essentially evaluates whether ML algorithms can support a precision-medicine approach to blood glucose control by better selecting likely responders to a lifestyle intervention. After training 11 different supervised learning algorithms and a standard logistic regression (LR) model, only a Random Forest (RF) achieved a higher correct classification fraction (CCF) (68.5%), than a completely naive model that simply classified all subjects according to the majority class (68.0%) which were the non-responders. Thus, only an additional 4 from 734 subjects (0.5%) were correctly classified by employing a RF algorithm. At face value, this appears very scant reward for the considerable time, effort and cost for deploying an algorithm that requires collating data on 59 variables taken from blood and urine examinations, abdominal ultrasounds, vital signs, indirect calorimetry, bioimpedance analysis, anthropometry, demographic data and medical history. However, classification accuracy on its own offers only a limited story when a more subtle and nuanced evaluation of performance is essential.

When embarking on this or similar studies, it would be wise to first consider what the potential for improved prediction might be for a ML algorithm versus a standard LR model. Several important factors can influence the outcome to this question including the true underlying nature of the predictor-outcome associations, the choice or availability of features used to build the model, and the range of ML algorithms deployed. If the underlying predictor-outcome associations are essentially linear and do not contain interactions, then a LR model which avoids unnecessary complexity is likely to perform either similarly well or even better than most ML models. But since we usually don’t know the exact complexity of the patterns within our data, a range of algorithms with varying underlying architectures are trialled; here a total of 11 different algorithms were assessed and it is worth briefly considering the main features that influence their performance and why they likely performed better or worse than the LR model. First the LR-based LASSO and Ridge regression models add an additional penalty term to a standard LR model to avoid over-fitting. Tree-based ML models (decision trees, RFs, bagged trees) perform well when important interactions do exist because they inherently incorporate interactions, and neural networks that incorporate non-linearities via hidden layers and activation functions, may easily outperform more basic models that assume linearity and homogeneous effects across sub-groups. This may help explain the much improved performances seen in this study for the above algorithms compared to the LR model. Support vector classifiers aim to define either linear or non-linear boundaries between the outcome classes, whilst discriminant analysis techniques and principal component analyses reduce dimensionality by transforming the data into a smaller number of features that are based on weighted combinations of the original features. However, this most often comes at a cost, especially if only the first two PCA components are used, which here resulted in substantially lower performance for each model compared to using the full dataset. It also explains why the gains in performance for Linear discriminant analysis (LDA) and Quadratic discriminant analysis (QDA) when using the full-feature datasets were relatively less than for other algorithms. The K-NN algorithm that relies on estimating the similarity of subjects is usually not the preferred algorithm when the importance of individual features varies widely since K-NN includes all features and assigns them equal weighting when calculating the patient similarity, diluting the effectiveness of the more important features. The elegant feature importance plots illustrate how certain features including HDL cholesterol, Hgb, urea, stature and white blood cells were considerably more informative than others, and offer an explanation as to why the K-NN algorithm likely performed poorly. In summary, model performance will depend both on the nature of the data, its richness, and the underlying characteristics that define each modelling approach. Here, the richness of data (59 features), their many potential interactions, and a large suite of algorithms that rely on very differing loss functions and methods, maximises the opportunity for improved performances from a ML-based approach compared to a LR model.

Next, we must decide, on what basis should we select one of our models as being the final and “best” performing model? A requirement of the new TRIPOD-AI reporting guidelines (item 12e) for the performance of regression-based and machine learning based prediction models [6] is the specification of all measures and plots used (and their rationale) to evaluate model performance. It is also recommended that a combination of multiple metrics be used and model performance be interpreted holistically since “*using only a subset of metrics could give a false impression of a model’s actual performance, and in turn, yield unexpected results when deployed to a clinical setting*” [7]. Foppiani et al. chose CCF as their primary accuracy metric for model selection on the basis that to offer any clinical utility whilst also ensuring that economic resources are conserved, a prediction model should achieve an overall classification accuracy that matches the historical failure rate (percentage of non-responders). However, CCF can often be a poor first choice measure for assessing predictive performance since it is considerably misleading where there are meaningful differences in class proportions. Simply assigning all samples to the most prevalent class to provide a “naïve” classifier can result in impressive accuracy scores if the imbalance is sufficiently large. Second, and a common trap to avoid when using medical data, only if the costs and rewards for not correctly identifying each outcome class can be considered completely equivalent does it make sense to use CCF alone as the basis for model selection. Specifically, whilst it is certainly important to avoid wasted resources, it is surely even more important to avoid missing the potential clinical and economic benefits of offering first-line therapy, for which a sizeable thirty-two percent of subjects responded.

Table 1 details the retrospectively constructed 2 × 2 confusion matrices for a naïve model and for the RF algorithm. The RF predicted 438 of the 499 non-responders and 65 of the 235 responders. These 65 true negatives will gain a tremendous benefit by being able to control their blood glucose using first-line treatment and can avoid a potential lifetime of costly treatment and associated detrimental side-effects. The only downside for the RF compared to the naïve classifier is that the RF misclassified 61 subjects as responders, but who ultimately did not achieve their target glucose levels using the 12-month intervention. However, if such an algorithm were employed in practice for first-line therapy selection, these patients would at least have been given the opportunity to trial the intervention and would therefore likely be more accepting of treatment escalation after 12 months. Contrast this to the only upside of making the sweeping assumption that no subjects will respond which is to conserve resources (data collation and deployment of the algorithm) and offer the 61 non-responding subjects treatment escalation 12-months sooner. Whilst selecting CCF as the primary metric was maybe therefore not ideal in this setting, Foppiani et al. did however appropriately also include AUC as a supporting metric. Here is where the algorithms can be seen to shine, with the RF easily outperforming both logistic regression (AUC = 0.687 versus 0.615) and the naïve classifier (AUC = 0.5 assuming 100% sensitivity and 0% specificity). A Boosted Trees algorithm performed better still (AUC = 0.702). Model performance based on discrimination (AUC) is one of several more informative metrics than CCF, including sensitivity (also known as recall), specificity, and positive predictive value (also known as precision). Since AUC, may also be a sub-optimal metric to present for clinical applications when a class imbalance exists [8], the related precision-recall curve (PRC) metric should also be considered [9].

**Table 1:** Confusion matrices for the Random Forest (RF) classifier versus a Naïve classifier using data from Foppiani et al.

	Predicted Non-responder	Predicted Responder	Total
**RF classifier**			
True non-responder	438 (True positive)	**61 (False positive)**	499
True responder	**170 (False negative)**	65 (True negative)	235
Total	608	126	734
**Naïve model**			
True non-responder	499 (True positive)	**0 (False positive)**	499
True responder	**235 (False negative)**	0 (True negative)	235
Total	734	0	734

Accuracy: Naïve model: 499/734 = 0.68.0%. RF: (438 + 65)/734 = 68.5%

Sensitivity: Naïve model: 499/499 = 100.0%. RF: 438/499 = 87.8%

Specificity: Naïve model: 0.0%. RF: 65/235 = 27.7%.

Positive predictive value: Naïve model 499/734 = 68.0%. RF = 438/608 = 72.0%

Negative predictive value: Naïve model: 0/0 = 0.0%. RF = 65/126 = 52.0%

In conclusion, under the right conditions, ML algorithms can certainly outperform less complex regression-based models. However, appropriate evaluation of model performance is essential; using CCF alone and comparing this to the percentage for the majority class (the naïve classifier) can easily present a cup half-empty view of model performance when either a moderate or large class imbalance exists, and especially when the economic and clinical costs associated with each type of misclassification are unequal. Instead, metrics for evaluation should focus on those that can consider these costs separately; reporting several different metrics including AUC and the PRC should become standard practice to ensure adequate guidance for final model selection. To do otherwise risks forming a biased and overly pessimistic view of ML model performance; one which may ultimately result in missed opportunities for precision-medicine and clinical decision support.
